# Assessment of Inter and Intra-atrial Asynchrony in Patients with Systolic Heart Failure Using Velocity Vector Imaging

**DOI:** 10.5812/cardiovascmed.10332

**Published:** 2013-07-31

**Authors:** Maryam Esmaeilzadeh, Marzieh Nikparvar, Majid Maleki, Feridoun Noohi, Zahra Ojaghi Haghighi, Niloufar Samiei, Paridokht Nakhostin-Davari, Hooman Bakhshandeh

**Affiliations:** 1 Echocardiography Research Center, Rajaie Cardiovascular Medical and Research Center, Iran University of Medical Sciences, Tehran, IR Iran; 2 Cardiovascular Research Center, Hormozgan University of Medical Sciences, Bandar Abbas, IR Iran; 3 Rajaie Cardiovascular Medical and Research Center, Iran University of Medical Sciences, Tehran, IR Iran

**Keywords:** Strain, Heart Failure, Asynchrony

## Abstract

**Background:**

According to previous studies on the deformation properties of the left atrium, the systolic strain and strain rates represent the atrial reservoir function and the early and late diastolic strain rates show the conduit and booster functions, respectively.

**Objectives:**

We sought to evaluate the intra and interatrial asynchrony using strain/strain rate imaging in systolic heart failure patients.

**Patients and Methods:**

Twenty five patients with systolic heart failure (LVEF ≤ 40%) were enrolled into the study. Asynchrony quantifications were performed according to the standard deviation of time-to peak (TP-SD) of deformation of three segments manually located along the perimeter of the left atrium free wall, right atrium free wall and interatrial septum, as imaged in an apical four-chamber view. We also calculated classic echocardiography parameters such as LV end-diastolic dimension index, LA volume index, RA area, as well as deceleration time (DT) on transmitral pulsed wave Doppler and E/E’ ratio on mitral annular tissue Doppler imaging.

**Results:**

In heart failure patients either inter or intra-atrial asynchrony were far more common in comparison with normal subjects (P=0.008 and P=0.007 respectively).

**Conclusions:**

Left ventricular systolic heart failure, may result in inter and intra-atrial asynchrony even in clinically stable patients without significant pulmonary hypertension and diastolic dysfunction.

## 1. Background

Structural heart disease and heart failure (HF) can lead to electrical and structural remodeling of the atria (1). As a result of fibrosis, change in atrial size, increased anisotropy, cellular uncoupling and dispersion of refractoriness alteration in atrial conduction may occur (1, 2). Atrial fibrosis is a hallmark of structural remodeling that contributes to the AF substrate and hence cerebral ischemic events and an increased risk of death (3-5). The left atrium (LA) acts as a booster pump during the late ventricular diastole and as a passive conduit during early ventricular diastole and diastasis (3).

Although LA function can be assessed by echocardiography, using Doppler analysis of trasmitral and pulmonary vein flow and tissue Doppler assessment of myocardial velocities (1) yet , assessment of LA deformation profiles should be obtained using Doppler-derived strain imaging which has been recently proposed as an alternative method for LA function (3) and evaluation of inter and intra-atrial conduction delay in heart failure (1, 6). However, this approach is limited by suboptimal reproducibility, angle dependency and confounding effect of noise artifacts (3, 5, 7). Speckle tracking echocardiography (STE) is a novel non-Doppler based method for angle-independent and objective quantification of myocardial deformation from standard two-dimensional images (5, 7). The recent availability of speckle tracking echocardiography has enhanced our ability to assess regional ventricular and atrial strain to evaluate ventricular and atrial synchrony and performance (8-10). In a recent study LA asynchrony was evaluated by measurement of time interval from peak of R (as reference point) to peak of left atrial positive strain before and after AF cardioversion using Speckle tracking echocardiography (STE) (7).

## 2. Objectives

In the present study, we have used this novel tool for studying LA cavity characteristics in a group of patients with systolic heart failure. We used speckle tracking echocardiography to evaluate inter and intra-atrial asynchrony in patients with systolic heart failure.

## 3. Materials and Methods

The study population consisted of 25 patients (mean age = 51 ± 13.3 years), with LV systolic dysfunction (LV EF ≤ 40%), which were referred for routine diagnostic or follow up echocardiography and twenty-four healthy adult subjects, referred for a diagnostic examination or checkup (mean age: 41.5 ± 14.5 years). All control subjects had unremarkable clinical history and normal findings on physical examination. The etiology of systolic dysfunction was either ischemic or nonischemic. The most common presenting symptom was dyspnea on exertion, functional NYHA class II – III. All subjects were in sinus rhythm and hemodynamically stable, without any significant valvular disease.

### 3.1. Standard Echocardiography

Echocardiography studies were performed using Mylab 60, Esaote, S.P, A-Italy. Subjects were studied in the left lateral recumbent position. Measurement of left ventricular (LV) dimension, left atrial (LA) volume and right atrial (RA) area were made in accordance to current recommendations of the American society of echocardiography. LVEF was estimated by visual assessment and Simpson's rule. The ratio between peak early diastolic filling velocities (E) and annular early diastolic velocity (E'), late diastolic filling velocity (A), deceleration time, systolic pulmonary artery pressure and right ventricular function were determined by standard Doppler and Doppler tissue imaging. LA volume and LV end diastolic dimension were subsequently indexed to body surface area.

### 3.2. Echocardiographic Measurements

All echocardiographic studies were performed with the subject lying in the left lateral decubitus position, obtaining complete 2D-echocardiographic color Doppler examination with a 3.5 MHz variable-frequency harmonic phased-array transducer. The LA volume was calculated according to the formula: 8/3p [(LA area in apical 42 chamber x LA area in apical two-chamber)/LA length] (11). LV global systolic function was evaluated by calculating ventricular volumes (end-diastolic volume, EDV; end-systolic volume, ESV), obtained using real-time three-apical longitudinal planes, followed by manual tracing of the endocardial border with built-in software. LV EF was computed as usual. LA volumes, together with LV EDD were indexed to body surface area. Mitral deceleration time (DT) was also measured as the time interval between peak E wave and the zero intercept of the deceleration profile.

### 3.3. Velocity Vector Imaging

Velocity Vector Imaging (VVI) is a state-of-the-art technology that enables clinicians to examine the mechanical functions of the myocardium to determine myocardial dysfunctions and dyssynchrony (12-15). It uses individual vectors to display direction and relative velocity of frame-to-frame tissue movement, delivering motion measurement at any point in the cardiac cycle. VVI is a unique technique with complex signal processing but an easy assessment method for the time-volume curves which enables visualization, measurement and display of myocardial mechanics (16). It requires only a single frame tracing of the endocardial border to extract quantitative time-motion and volume data. Not only the feasibility of the currently available speckle-tracking echocardiographic strain technique (2D STE and VVI) but also the agreement between the two techniques have been shown (17). For VVI analysis, apical four chamber view images were obtained using conventional two-dimensional gray scale echocardiography during breath hold and with a stable ECG recording.

Particular attention was paid to obtain an adequate gray scale in each view and to avoid foreshortening of the left atrium, allowing a more reliable delineation of the atrial endocardial border. Three consecutive heart cycles were recorded and analyzed. Recordings were processed off line by a single experienced echocardiographer using a commercially available semi-automated two-dimensional strain software (Mylab 60, Esaote, S. p, A-Italy).

### 3.4. Atrial Asynchrony Quantitation

**Figure 1.**
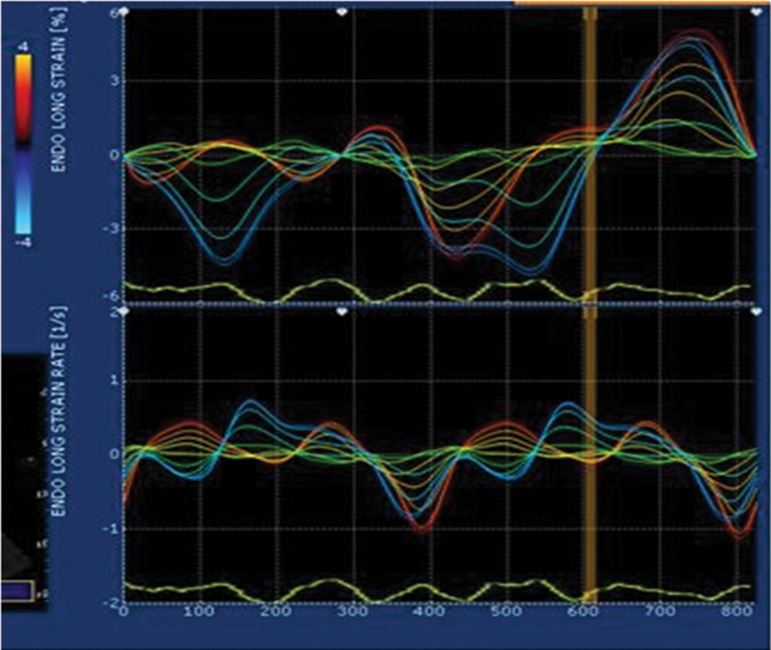
Atrial Strain and Strain Rate Curves of a Normal Subject

Left and right atria endocardial surfaces were manually traced in four chamber views in end systole and the resulting tracking quality for each segment was automatically scored as either acceptable or non-acceptable with the possibility of further correction. Lastly, strain curves were generated for each atrial segment by the software (Figure 1).

**Figure 2.**
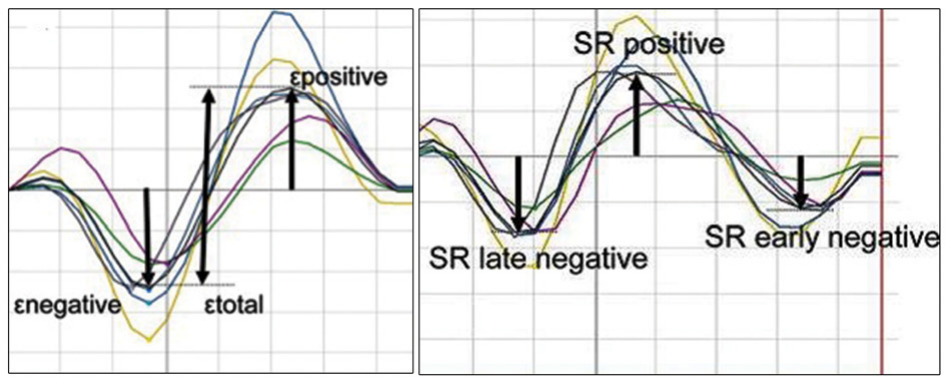
Schematic Atrial Strain (Left) and Strain Rate (Right) Curves

Peak R wave in ECG monitoring was the default reference point. We also considered the onset of P wave on the recorded electrocardiogram as the second reference point. Peak atrial longitudinal strain (PALS) was measured in LA segments at a distance of 0.5 - 1 cm from mitral annulus in four chambers for septal and lateral walls and 0.5- 1 cm below tricuspid annulus on right atrial (RA) free wall. First negative deflection of LA strain and strain rate curves (Figure 2) after P wave in same locations on three points of LA free wall, interatrial septum and RA free wall was considered as a wave.

**Figure 3.**
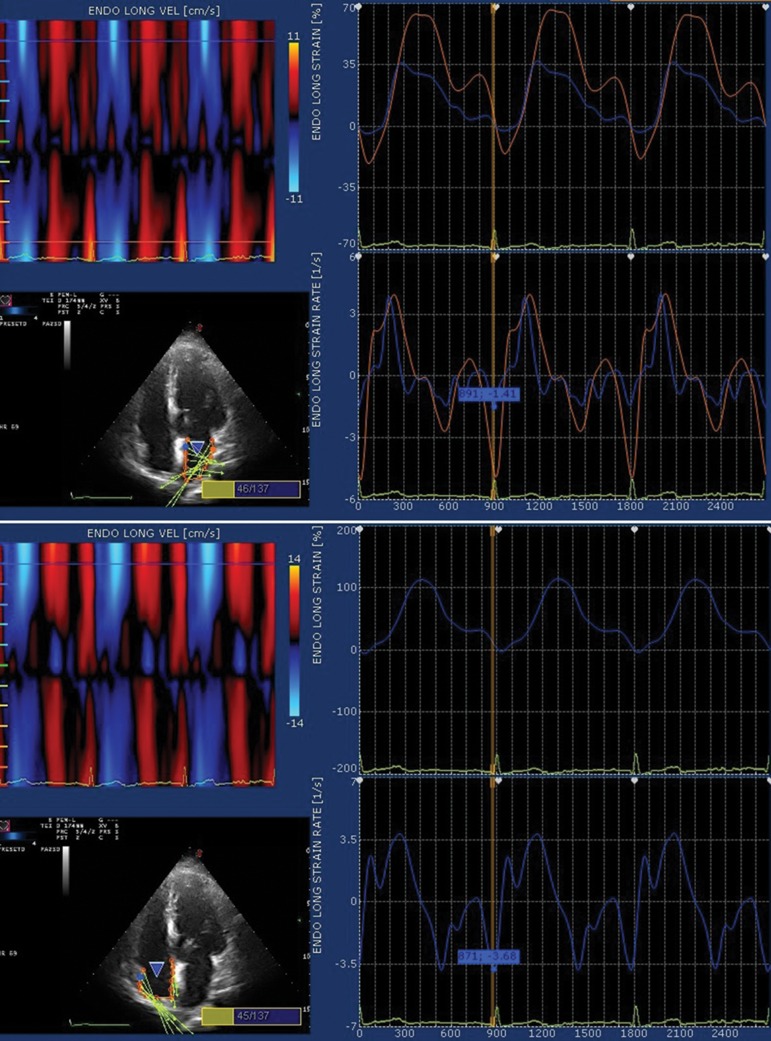
Evaluation of Atrial Deformation According to the Speckle-tracking Technique. Left Atrial (top) and Right Atrial (bottom) Endocardial Contour is Traced in a Four-chamber View

The time difference from onset of P wave to nadir of a wave at the RA free wall (P-RA), interatrial septum (P-IAS), and LA free wall (P-LA), were defined as time to atrial activation. The time difference from peak R wave to nadir of A wave at the RA free wall (R-RA), interatrial septum (R-IAS) and lateral LA wall (R-LA), were also defined ( 1 ). These time intervals were corrected by dividing them by the square root of RR interval in the same view (Figure 3).

### 3.5. Reproducibility

To assess the reproducibility of intra-atrial and interatrial asynchrony, five subjects were randomly selected and analyzed. Bland-Altman analysis was performed to evaluate the inter and intra-observer variability by repeating the analysis 1 week later by the same observer and a second observer and this resulted an intra-observer variability of 1.1% and an inter-observer variability of 2.2%.

### 3.6. Statistical Analysis

Data are expressed as mean ± SD for interval and count(%) for categorical variables. association between asynchrony and study groups was investigated by Student’s t test and between asynchrony and other interval variables by Pearson’s correlation coefficients. A P-value < 0.05 was considered to be significant. Statistical analysis was performed using the SPSS 16 for Windows (SPSS Inc.,Chicago, Illinois).

## 4. Results

Baseline echocardiographic and general characteristic data are shown in Table 1.

**Table 1.** Baseline Characteristics in Normal and Patient Groups

Variable	Patient (n = 25)	Control (n = 24)	P Value
**Age, y**	51 ± 13.3	41 ± 15.4	0.007
**Gender, No (%)**			
Female	6 (24%)	15 (62.5%)	0.028
Male	19 (76%)	9 (37.5%)	
**BSA^a^, m^2^**	1.79 ± 0.17	1.7 ± 0.13	> 0.05
**LV^a^ejection fraction (%)**	24.8 ± 7.2	55.8 ± 1.9	0.000
**LVEDDI^a^, cm/m^2^**	3.33 ± 0.52	2.68 ± 0.2	0.000
**LAVI^a^,cm^3^/m^2^**	29.29 ± 10.9	22.7 ± 4.7	0.008
**Right atrial area, cm^2^**	12.8 ± 2.8	11.2 ± 2.1	> 0.05
**Deceleration time, ms**	216.5 ± 68	192 ± 40	> 0.05
**Mitral E/E’ ratio**	9.5 ± 3.87	7.5 ± 2.37	0.53

^a^ Abbreviations: LV, left ventricle; LVEDDI, left ventricle end-diastolic volume; LAVI, left atrium volume index; BSA, body surface area

**Figure 4.**
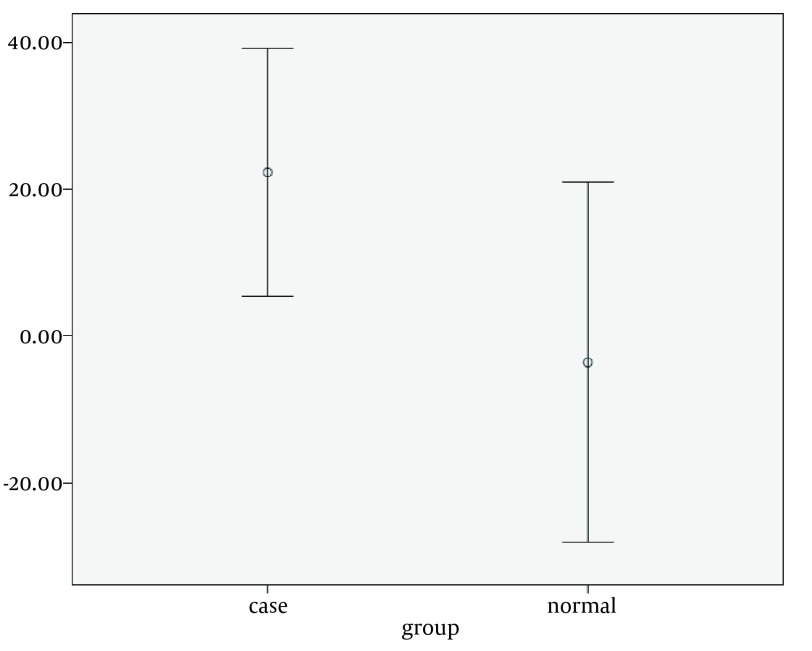
Comparison of Inter-atrial Asynchrony by Strain Rate Measurements in Heart Failure Patients and Control Group

There was a significant difference between time to onset of P wave and onset of atrial LA strain and strain rate curves in heart failure patients (249 ± 56 versus 214 ± 30 msec, P =0.009 and 191±45 versus 157 ± 26 msec, P = 0.003 respectively) (Figure 4). A significant difference was found between time to onset of P wave and onset of interatrial septum strain curves in heart failure patients (149 ± 27 versus 198 ± 26 msec, P = 0.048) (Table 2).

**Table 2.** Time to Peak of Atrial Contraction in Strain and Strain Rate Imaging

Deformation Indexes	Patients (n = 25)	Control (n = 25)	P Value
**(P-RA)^a^ msec**	217 ± 51	207 ± 42	> 0.05
**(RA) strain rate**	167 ± 49	161 ± 34	> 0.05
**(P-LA)^a^ msec**	249 ±56	214 ± 30	0.009
**(P-LA) strain rate**	191 ± 45	157 ± 26	0.003
**(P-IAS)^a^ msec**	278 ± 54	198 ± 26	0.048
**(P-IAS) strain rate**	157 ± 47	149 ± 27	> 0.05
**(R-LA)^a^corrected strain**	30.6 ± 3.4	30.9 ± 2.6	> 0.05
**(R-LA) corrected strain rate**	28.7 ± 3.14	28.71 ± 2.2	> 0.05
**(R-IAS)^a^corrected strain**	29.3 ±3.4	30.39 ± 2.5	> 0.05
**(R-IAS) corrected-strain rate**	26.84 ±3.75	28.28 ± 2	> 0.05

^a^ Abbreviations: P-RA, the time difference from P wave to pre atrial contraction strain of right atrium; P-LA, the time difference from P wave to pre atrial contraction strain of left atrium; P-IAS, the time difference from P wave to pre atrial contraction strain of interatrial septum; R-RA, the time difference from R wave to pre atrial contraction strain of right atrium; R-LA: the time difference from R wave to pre atrial contraction strain of left atrium, R-IAS: the time difference from R wave to pre atrial contraction strain of interatrial septum

Strain rate measures showed significant difference in intra-atrial synchrony [(P-LA)-(P-IAS): time difference between time to onset of P wave and onset of LA free wall and interatrial septum strain rate)] in heart failure patients (35 ± 47 msec versus 22±37msec, P = 0.014). Inter atrial asynchrony C SR in case group was significantly different from the control group, P = 0.007. LA asynchrony SR and LA asynchrony CSR in case group was significantly different from the control group (P = 0.008 and P = 0.007 respectively), (Table 3). 

**Table 3.** Inter and Intra Atrial Asynchrony in PatientsVersus Normal Control

Asynchrony	Patients	Control	P Value
**RAaasynchrony S**	32.52 ± 27.3	31.8 ± 28.6	> 0.05
**RA ** ^**a**^ **synchrony SR**	28 ± 25.4	27.4 ± 21.8	> 0.05
**LA^a^asynchrony S**	43.56 ± 36.3	29.2 ± 26.8	> 0.05
**LA asynchrony SR**	45.6 ± 22.7	26.6 ± 19.85	0.008
**Inter-atrial asynchrony S^a^**	40.34 ± 32.37	40.82 ± 37.5	> 0.05
**Inter-atrial asynchrony SR^a^**	35.8 ± 24.7	33.8 ± 32.5	> 0.05
**RA asynchrony CS^a^**	1.6 ± 1.37	1.84 ± 1.7	> 0.05
**RA asynchrony CSR** ^**a**^	5.8 ± 18.5	1.8 ± 1.4	> 0.05
**LA asynchrony CS**	1.7 ± 1.3	1.1 ± 1	> 0.05
**LA asynchrony CSR**	2.15 ± 2.3	0.9 ± 0.79	0.007
**Inter-atrial asynchrony CS**	1.77 ± 1.42	1.99 ± 1.6	> 0.05
**Inter-atrial asynchrony CSR**	2.15 ± 2.3	0.9 ± 0.78	0.007

^a^ Abbreviations: RA, right atrium; LA, left atrium; S, strain; SR, strain rate; CS, corrected strain; CSR, corrected strain rate

Strain rate measures also showed significant differences in interatrial synchrony [(P-LA)-(P-RA): time difference between time to onset of P wave and onset of LA free wall and RA free wall strain rate)] in heart failure patients (191 ± 45 msec versus 167 ± 49 msec, P = 0.02), (Figure 4).

**Figure 5.**
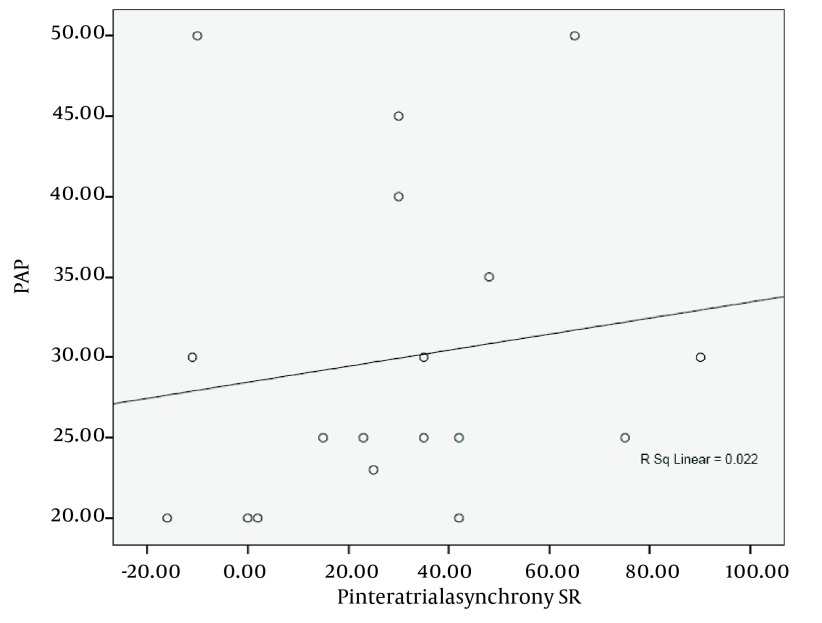
Correlation Between Corrected Interatrial Asynchrony (Strain Rate Measurements) and Systolic Pulmonary Artery Pressure, P = 0.022, r = 0.5

Corrected inter-atrial asynchrony and LA asynchrony by strain rate study was significantly different from control group (both P = 0.007), (Table 3). There was a correlation between intra and interatrial asynchrony with general characteristics and baseline echocardiographic variables. Strain rate measures showed a moderate direct correlation between corrected interatrial asynchrony with mean systolic pulmonary arterial pressure in HF patients (P = 0.022, r = 0.5, Figure 5). Also weak negative but significant correlations were found between corrected intra and interatrial asynchrony (strain rate imaging) and intra-atrial asynchrony (strain imaging) with LVEF (P = 0.002, r = −0.3). There was a weak positive correlation between indexed LA volume and interatrial asynchrony indicated by the strain imaging study (P = 0.011, r = 0.325). There was a moderate direct correlation between age and corrected inter and intra-atrial asynchrony either by strain or strain rate imaging (r = 0.5).

## 5. Discussion

In this study we analyzed intra and inter-atrial asynchrony using a novel speckle tracking index in patients with advanced systolic heart failure. The results of our study suggest that 2D strain is currently available as a non-invasive tool to quantify atrial deformation and asynchrony. Since heart failure is associated with atrial conduction delay, strain contributes to a better definition of the mechanical characteristics as is known for the ventricle. In this study 2D strain using velocity vector imaging was used to evaluate intra- and inter-atrial asynchrony in patients with HF and controls. In a study by Beeumen et al. (1) atrial asynchrony was evaluated for 23 controls (65 ± 13 years), 29 patients with structural heart disease without HF (68 ± 9 years), and 29 patients with HF (67 ± 9 years) using color tissue Doppler imaging. Asynchrony was defined as the differences between P-IAS and P-RA (RA asynchrony), P-LA and P-IAS (LA asynchrony), and P-LA and P-RA (inter-atrial asynchrony).

There are some differences between our findings and theirs. They reported a significant increase in RA asynchrony in HF patients compared with controls and patients without HF (30 ± 21 versus 12 ± 13 and 14 ± 15 ms, P < 0.001), but in our study RA asynchrony was not different between patients and control subjects (28 ± 25.4 versus 27.4±21.8 msec, P > 0.05). On the contrary, they were not be able to show a significant difference in LA asynchrony (19 ± 26 versus 25 ± 13 versus 25 ± 14 msec, P > 0.05), although we found a significant LA asynchrony in our patients (45.6 ± 22.7 versus 26.6 ± 19.85, P = 0.008). Although in their study inter-atrial asynchrony was significantly higher (49 ± 24 versus 37 ± 9 and 39 ± 17 msec, P = 0.04), in ours it was significant just in terms of CSR (2.15 ± 2.3 versus 0.9 ± 0.78 1/s, P = 0.007).

In their study, only the correlations between RA asynchrony with the ejection fraction was significant (P < 0.001, r = −0.4), however, we found a weak negative but significant correlation between corrected intra and inter-atrial asynchrony (strain rate imaging) and intra-atrial asynchrony (strain imaging) with LVEF (P = 0.002, r = −0.3). Our study showed that atrial asynchrony assessment using VVI is feasible in heart failure patients in clinical practice and some degree of either LA asynchrony and or inter-atrial asynchrony may present in patients with systolic heart failure. In addition, it is unknown that atrial asynchrony responds to cardiac resynchronization therapy.

In a recent comparative study for assessment of left atrial deformation and synchrony in healthy subjects and patients with atrial fibrillation (AF) using three-dimensional speckle-tracking echocardiography Mochizuki et.al revealed that both LA longitudinal strain (LS) and circumferential strain (CS) (12.0 ± 4.1 versus 7.0 ± 3.9, P after Bonferroni’s correction < 0.05 and 22.8 ± 8.1 versus 10.7 ± 7.2, P after Bonferroni’s correction < 0.05 respectively) and also standard deviation of either left atrial systolic longitudinal strain ( LSs) or pre atrial contraction longitudinal strain (LSa) were reduced in patients with paroxysmal AF compared with controls (SD LSs (%): 9.9 ± 5.8 versus 16.1 ± 6.8, P after Bonferroni’s correction < 0.05, SD LSa (%): 20.9 ± 10.9 versus 26.4 ± 8.8), and further reduction of all parameters were observed in patients with permanent AF.(18). Comparison of LA strain and synchrony between controls and patients with paroxysmal AF showed a significant dyssynchrony in patients with LA dilation (SD LSs (%): 9.9 ± 5.8 vesus 16.3 ± 7.6, P after Bonferroni’s correction < 0.05, SD LSa (%): 20.9 ± 10.9 versus 26.4 ± 8.8). In addition using two-dimensional STE, longitudinal and circumferential LA strain during ventricular systole (LSs) were significantly smaller in patients with paroxysmal AF than in controls (23.8 ± 8.6 versus 32.6 ± 6.5) (18). In this study, the amount of strain value (%) was assessed as a criterion for LA function and synchrony, however, in our study, we measured the time interval (msec) from the onset of P wave to the onset of pre atrial contraction strain and strain rate curve which seemed to be a far better marker of asynchrony or electromechanical delay in both atria.

### 5.1. Clinical Implications

The present study showed that VVI enables the comprehensive assessment of LA synchrony in patients with systolic heart failure with excellent reproducibility. It has been previously shown that in patients with AF, reduced LA strain determined by 2D STE is correlated with fibrosis in LA wall demonstrated by cardiac magnetic resonance imaging, (2) histologically proven left atrial myocardial fibrosis in patients with mitral valve disease, (19) CHADS2 risk score (20) and history of stroke (21) in patients with persistent AF. Besides these, cardiogenic stroke (21), prognosis in patients with acute myocardial infarctions (21) and cardiovascular diseases (22) and recurrence of AF after pulmonary venous isolation (23) have been reported to be predictable from LA strain (either determined by tissue Doppler imaging or 2D STE).

Although, an improvement in LA atrial strain following cardiac resynchronization therapy in HF patients has been shown (24), further investigation on this issue should be mandatory. Ongoing studies on atrial dynamics and functional remodeling will enable us to develop more effective strategies for the treatment and prevention of AF, HF, and stroke (24). Atrial asynchrony assessment using VVI is feasible in heart failure patients in clinical practice. There are some degree of LA asynchrony and inter-atrial asynchrony in patients with systolic heart failure, even in stable patients and in those without significant pulmonary hypertension.

### 5.2. Study Limitations

2D speckle tracking of the atria is more difficult and time consuming than those of ventricles. Both the left and right atria are farther from the transducer in the apical views, and their myocardium is thinner and brighter than the ventricular myocardium, so fewer speckles could be tracked. Another limitation was the ability to only assess longitudinal function.
